# Wedge prism approach for simultaneous multichannel microscopy

**DOI:** 10.1038/s41598-019-53581-9

**Published:** 2019-11-28

**Authors:** Hanna Cai, Yao L. Wang, Richard T. Wainner, Nicusor V. Iftimia, Christopher V. Gabel, Samuel H. Chung

**Affiliations:** 1 GAMDAN Optics Inc., 2362 Qume Drive, Suite B, San Jose, CA 95131 USA; 2 Northeastern University, Department of Bioengineering, 360 Huntington Avenue, Boston, MA 02115 USA; 3 Physical Sciences Inc., 20 New England Business Center, Andover, MA 01810 USA; 4 Boston University School of Medicine, Department of Physiology and Biophysics, 700 Albany Street, Boston, MA 02118 USA; 5 Boston University, Boston University Photonics Center, 8 Saint Mary’s Street, Boston, MA 02215 USA; 6 Boston University School of Medicine, Department of Pharmacology and Experimental Therapeutics, 72 East Concord, St. Boston, MA 02118 USA

**Keywords:** Ca2+ imaging, Wide-field fluorescence microscopy, Optical imaging, Fluorescence imaging

## Abstract

Multichannel (multicolor) imaging has become a powerful technique in biology research for performing *in vivo* neuronal calcium imaging, colocalization of fluorescent labels, non-invasive pH measurement, and other procedures. We describe a novel add-on approach for simultaneous multichannel optical microscopy based on simple wedge prisms. Our device requires no alignment and is simple, robust, user-friendly, and less expensive than current commercial instruments based on switchable filters or dual-view strategies. Point spread function measurements and simulations in Zemax indicate a reduction in resolution in the direction orthogonal to the wedge interface and in the axial direction, without introducing aberration. These effects depend on the objective utilized and are most significant near the periphery of the field of view. We tested a two-channel device on *C. elegans* neurons *in vivo* and demonstrated comparable signals to a conventional dual-view instrument. We also tested a four-channel device on fixed chick embryo Brainbow samples and identified individual neurons by their spectra without extensive image postprocessing. Therefore, we believe that this technology has the potential for broad use in microscopy.

## Introduction

Simultaneous multichannel (*i.e*., multicolor) microscopy techniques are finding ever greater application in biology as noninvasive measurements become more attainable and desirable. Example techniques include *in vivo* ratiometric neuronal calcium imaging, colocalization of fluorescent labels, and non-invasive pH measurement. Monochrome cameras are the standard in modern light microscopy since they are significantly more sensitive and less expensive than color cameras. Thus, to maximize flexibility, multichannel approaches often convert existing single channel microscopes to perform multichannel imaging. There are two general strategies: fast-switchable filters split observation time between channels, or mirrors separate and redirect light (*e.g*., dual-view)^1^. Devices based on these approaches suffer from a variety of limitations: they use fast moving parts that degrade, image channels non-simultaneously, require substantial non-user-friendly hardware additions, have difficulty imaging more channels, require precise alignment in space or time, or are expensive. By employing wedge prisms, our approach overcomes these limitations. In this paper, we present the theory behind our approach, its implementation, and the characterization of our devices, both theoretically and experimentally. We also demonstrate the imaging performance of our devices on biological samples.

## Wedge Device Operation

### General concept

**Figure 1:**
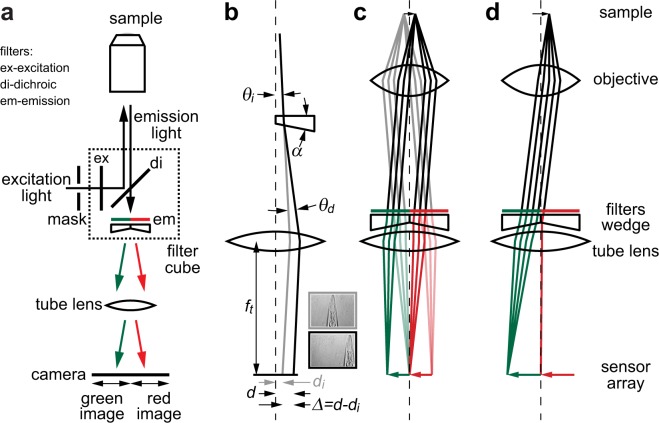
Wedge prisms permit simple multichannel imaging. (**a**) Optics and light beampaths for separating channels in an epifluorescence microscope. (**b**) Ray diagram of wedge deflecting single ray. Insets show undeflected (top) and 1°-deflected (bottom) brightfield images of *C. elegans* nose *in vivo*. (**c**) Ray diagram of wedges tiling channels side-by-side on CCD array. (**d**) When employing wedge, effective numerical aperture of points imaged near CCD center is reduced compared to points imaged near CCD edge.

Our novel approach uses wedge prisms to redirect the channels to separate areas of a single camera sensor array. We employ a charge-coupled device (CCD). As shown in Fig. 1a, multichannel emission light passes through side-by-side emission filters and forms parallel beamlets containing the two channels. Side-by-side wedge prisms, which have a small apex (or wedge) angle *α*, refract light at their faces and deflect the beamlets in opposite directions. The microscope tube lens images the channels on separate camera regions. Thus, by replacing a single emission filter in a filter cube, our novel device allows standard epifluorescence microscopes to perform simultaneous multichannel widefield imaging, with distinct advantages in cost, simplicity, robustness, and flexibility.

### Wedge effect on single ray

Figure 1b shows the path of a representative light ray of initial angle *θ*_*i*_ passing through a prism of apex angle *α* and refractive index $n={n}_{wedge}/{n}_{air}$. As derived in Supp. Note: Derivation of deflection angle, typical wedge prisms deflect paraxial rays by

$${\theta }_{d}\cong (n-1)\alpha .$$

This deflection angle is independent of *θ*_*i*_ in the paraxial approximation, which holds for the space between the objective and the camera. The tube lens converts the angular deflection *θ*_*d*_ into a lateral displacement at the camera of $\varDelta =d-{d}_{i}$, where *d* and *d*_*i*_ are the displaced and initial ray positions, respectively. As derived in Supp. Note: Derivation of image displacement for paraxial rays,

$$\varDelta \cong {f}_{t}\,\tan ({\theta }_{d}),$$

where *f*_*t*_ is the tube lens focal distance. Note that the lateral displacement is independent of the axial position of the wedge, so long as it resides between the objective and tube lens.

### Initial wedge testing and aberration calculations

We tested off-the-shelf wedges with *θ*_*d*_ = 1° and 0.5° using fluorescent beads (see Methods) and find that image displacement, *Δ*_*exp*_, matches the theoretical prediction, *Δ*_*theor*_, and is independent of the objective (see Table 1 and Fig. 1b inset). We also pursued a theoretical understanding of aberrations arising from a wedge approach (see also error calculations in the Supp. Notes). The largest effect is a chromatic aberration resulting from a wavelength-dependent refractive index (*i.e*., chromatic dispersion). Since channels are spatially separated, this is only an issue within each channel. Chromatic aberration leads to a wavelength-dependent change in the angle deflected and a lateral shift of the position on the camera sensor. The shift is in the direction of the wedge deflection. Table 2 calculates the displacement, *Δ*, for two coincident rays with wavelengths at the opposite ends of green and red channel bandwidths. The wedge angle for each of three substrates is set to make *Δ* = *L*/4 for λ = 500 nm, where *L* is the length of the camera sensor’s longer side. For our camera and BK7 wedges, the distance between the deflections corresponds to a small 1–2 pixel shift, leading to a degradation of the resolution in the direction of wedge deflection (see below). As described below, we can decrease the chromatic aberration by utilizing wedge substrates with reduced chromatic dispersion or employing an achromatic design with two compensating substrates, similar in principle to the achromatic doublet lens.

**Table 1:** Wedge testing.

*α* (deg)	*θ*_*d*_ (deg)	objective	*Δ*_*exp*_ (mm)	*Δ*_*theor*_ (mm)	% diff	*T*_*wedge*_*/T*_*dual-view*_
1.93	1.00	10x	3.55	3.49	1.7%	nt
1.93	1.00	20x	3.54	3.49	1.3%	nt
1.93	1.00	40x	3.55	3.49	1.6%	nt
1.93	1.00	60x	3.54	3.49	1.3%	nt
1.93	1.00	100x	3.54	3.49	1.5%	nt
1.24	0.64	10x	2.31	2.23	3.6%	0.58 ± 0.07
1.24	0.64	20x	2.32	2.23	3.7%	0.65 ± 0.10
1.24	0.64	40x	2.32	2.23	3.9%	0.96 ± 0.08
1.24	0.64	60x	2.32	2.23	3.9%	0.57 ± 0.06
0.97	0.50	60x	1.74	1.75	−0.1%	nt

% diff-percent difference, nt-not tested.

**Table 2:** Chromatic aberration calculations.

wedge substrate	channel	* λ* (nm)	*n* Sellmeier	*θ*_*d*_ (deg)	* Δ* (mm)	lateral shift (μm)	pixels shifted
BK7	green	500	1.5214	0.6430	2.245	12.5	1.9
		550	1.5185	0.6394	2.232		
	red	600	1.5163	0.6367	2.223	9.0	1.4
		660	1.5142	0.6341	2.214		
fused silica	green	500	1.4899	0.6430	2.245	9.5	1.5
		550	1.4878	0.6403	2.235		
	red	600	1.4862	0.6382	2.228	6.8	1.1
		660	1.4847	0.6362	2.221		
CaF_2_	green	500	1.4365	0.6430	2.245	8.5	1.3
		550	1.4348	0.6406	2.236		
	red	600	1.4336	0.6387	2.230	6.0	0.9
		660	1.4324	0.6370	2.224		

Chromatic aberration for green and red channels for given wedge substrates. We utilized BK7 substrates for this study.

### Two-channel operation

For two-channel imaging, we define the image displacement, *Δ* = ±*L*/4. This deflects light toward the centers of the two sensor halves and tiles the two channels on the sensor array, as depicted in Fig. 1c. Thus, $\alpha =\frac{1}{n-1}{\tan }^{-1}(\frac{L}{4{f}_{t}})$. For the two-channel portion of this study, we employed a Nikon Ti microscope (*f*_*t*_ = 200 mm), Andor Clara camera (*L* = 8.98 mm), and a custom two-channel wedge with *α* = 1.24° fabricated from BK7 (*n* = 1.51), a common laboratory glass substrate. The wedge device resides in the typical emission filter position of a filter cube. Typical microscope images overfill the camera sensor. We placed a rectangular mask in the field stop position of the excitation beam path to gate the sample area illuminated, limit the single-channel image size to half the sensor area, and prevent channel overlap. The field of view (FOV) of each channel is half of the original FOV with no change in magnification.

### Mirror vs. wedge optics

**Figure 2:**
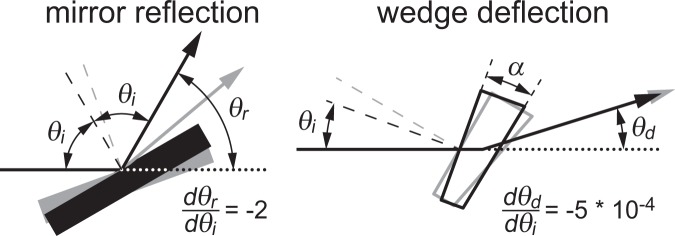
Wedge deflection is less sensitive than mirror reflection to errors in optic orientation.

Conventional dual-view instruments use mirrors and reflection to redirect light to the camera^1^. As shown in Fig. 2, an error in mirror orientation (grayed objects) changes both incident and reflected light angles, introducing twice this error in the reflected beam direction. Formally, ${\theta }_{r}=180-2{\theta }_{i},$ and thus, $\frac{d{\theta }_{r}}{d{\theta }_{i}}=-2$ for mirrors.

Wedge prisms redirect light by deflection (see Fig. 2). From equation (1) in the Supp. Note, the derivative of the deflection angle is $\frac{d{\theta }_{d}}{d{\theta }_{i}}=({n}^{2}-1)\alpha [\frac{{\theta }_{i}}{n}-\frac{\alpha }{2}]$. For our experiments, *n* = 1.51, *α* = 1.24°, and −0.64° < *θ*_*i*_ < 0.64°. Thus, max $\frac{d{\theta }_{d}}{d{\theta }_{i}}=-\,5\,\ast \,{10}^{-4}$ for a typical wedge setup and the introduced error in the deflected beam direction is only 5*10^−4^ times the error in wedge orientation. Errors in wedge orientation have orders of magnitude lower impact on image alignment compared to errors in mirror orientation. Thus, one key advantage of wedges compared to mirrors is that they eliminate the need for precise spatial alignment, allowing users to use our device by simply inserting it into the emission beam path. This makes the device more robust and easier to use.

### Advantages and disadvantages of wedge approach

The novel wedge approach features several advantages over existing multichannel instruments employing monochrome cameras. Unlike fast filter changers (*e.g*., Leica EL6000), that switch between filters to image channels sequentially, the wedge device records truly simultaneous, high-speed measurements without fast-moving, degrading parts or synchronization software. Unlike “dual-view” devices (*e.g*., Photometrics DV2) that also perform simultaneous multichannel imaging by tiling channels, the wedge approach is straightforwardly scalable to a larger number of channels without presenting any additional complexity to the user. Also, unlike the application of a Bayer mask to filter light to individual pixels, the wedge approach needs no access to the sensor array or alignment. Our device fits into a standard filter cube familiar to most users, is easily moved from the beampath if unused, has no moving parts, and requires no external footprint or special expertise to operate. When including all the additions required for converting a microscope from single channel to multichannel operation, our estimated profitable final price for a two-channel device is less than 1/3rd of existing commercial multichannel instruments. A previous study employed a wedge to separate channels^2^ but still redirected light by reflection and required precise alignment. Moreover, in some microscope setups this approach can lead to differences in optical path length and focal plane between the channels.

Despite its multiple advantages, our approach also has some inherent disadvantages compared to conventional instruments. The primary disadvantage is that our device utilizes only 50% of the available light for each channel because the emission light beam is split in half spatially to achieve two image paths. However, as described below, our tests indicate that the transmission through a microscope with the two-channel wedge device is 57–96% of the transmission through the microscope with a comparable dual-view instrument, presumably due to the fewer optics in our device (see Table 1). Moreover, recent enhancements in fluorophore design have greatly improved the intensity of sample emission and permit a variety of experiments *in vivo*. A second disadvantage is the addition of chromatic dispersion from utilizing wedges, which differentially refract various colors. As described above, this chromatic aberration leads to a small 1–2 pixel shift between light rays at opposite ends of our channel bandwidth. We discuss some mitigation strategies below.

## Wedge Device Fabrication, Characterization, and Demonstration

### Two-channel device

The two-channel wedge device deflects light toward the centers of the two CCD halves and consists of two identical custom-made semicircular wedges paired with semicircular filters. For each wedge, we ground down a blank 25 mm-diameter, 5 mm-thick BK7 window at a wedge angle of 1.24° to the axis. We confirmed the angle using a digital micrometer in conjunction with autocollimation. We polished the circular wedge faces to a flatness of better than *λ*/4 @ 633 nm. Employing an autocollimator we positioned the circular wedge and cut orthogonal to the gradient to create the semicircular wedges. The device’s filters originated from Chroma Technologies. We cut off-the-shelf 25 mm-diameter emission filters (red ET632-60 m and green ET520-40 m) through their center to generate the semicircular filters. We assembled the parts using rapid-curing epoxy to create the prototype. The filter cube also contained excitation and dichroic filters from multichannel filter set 59022.

**Figure 3:**
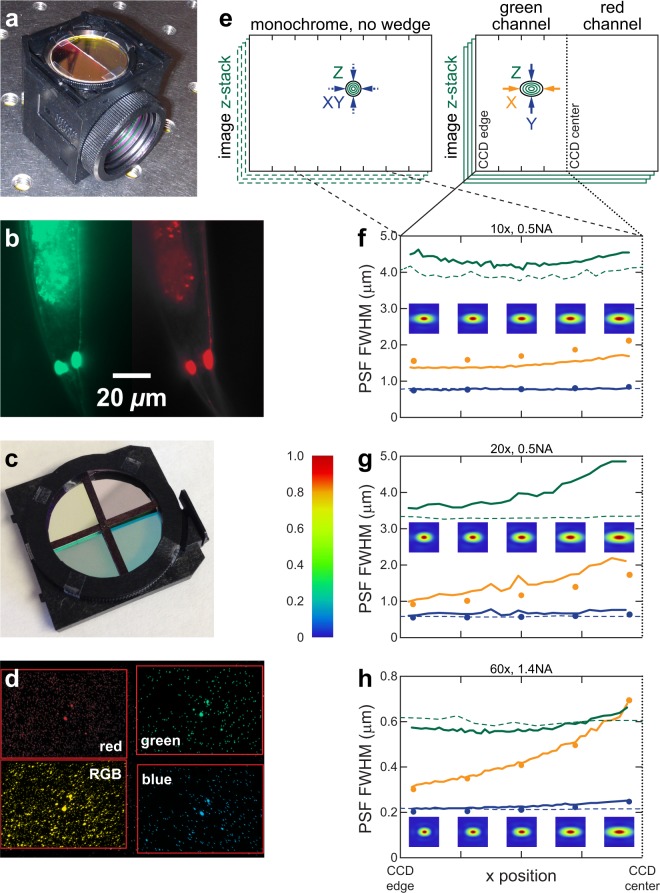
Wedge devices and testing. (**a**) Two-channel device installed in filter cube. (**b**) Two-channel false-color image of *C. elegans* neurons *in vivo*. (**c**) Four-channel device in emission filter housing. (**d**) Four-channel false-color image of red and blue-green beads. Red boxes outline channels. Lower left channel includes red, green, and blue light. (**e**) Diagram of green fluorescent bead PSF measurement without and with wedge device. (**f**–**h**) Resolution measurements under various objectives without (dashed) and with (solid) wedge device. FWHM values of PSF extent in x (yellow), y (blue), and z (green) are dependent on bead x position. Each point is the average of data from 40 beads. PSF FWHM from simulations indicated as points. Insets show simulated transverse PSF heatmaps at corresponding points.

Figure 3a shows an image of the custom two-channel wedge device in the emission filter position of the filter cube, while Fig. 3b shows a representative false-color image of *C. elegans* PLM neurons expressing green GCaMP and red mCherry genetically-encoded fluorophores. We characterized this device by imaging fluorescent beads and thin films of fluorescent dye (see Methods). While each channel of our device only uses 50% of the available light, under some objectives, the total transmission through a microscope with our two-channel device can be comparable to the total transmission through a microscope with a dual-view instrument. As shown in Table 1, the transmission with the two-channel wedge device, *T*_*wedge*_, is 57–96% of the transmission with a dual-view instrument, depending on the objective used. We attribute this lower than expected loss to the fewer optics producing back reflection and absorption in the wedge device compared to a dual-view instrument. Testing confirms an image displacement that matches the theoretical prediction (see Table 1). As further described in the Methods, we registered images by eye or a cross-correlation program and extracted horizontal and vertical translation parameters for use in future imaging. To orient the deflection direction along the long dimension of the camera array we manually adjusted the azimuthal angle of the entire device (rotating in the mount) by eye. Even so, the custom wedge device tiles the channels on the camera sensor very effectively, with only 3.8% of the sensor area not represented in both channels. Assuming a well-aligned microscope, precision alignment under mass production should eliminate the need for user alignment and further improve the performance.

We also characterized changes in imaging resolution from the addition of our wedge device to the microscope imaging beampath. Following established procedures^3^ we acquired 3D image stacks (*i.e*., z-stacks) of sub-diffraction limit 175-nm green fluorescent beads with and without our wedge device, and we utilized the PSFj software package^3^ to calculate the full width half maximum (FWHM) values of the point spread function (PSF), which we abbreviate as PSF_xyz_ below. The typical PSF for an ideal objective in a well-aligned microscope is isotropic in the lateral (xy) plane (see Fig. 3e, left side) and does not strongly depend on position in the image. The objectives and system demonstrate roughly ideal performance (see Fig. 3f–h, dashed lines).

The wedge deflects the center portion of the original FOV (see Fig. 3e, left) to the two halves of the CCD (see Fig. 3e, right). We measured the PSF on the left half of the CCD with green fluorescence. The PSF_y_ with the wedge (solid blue line) closely tracks the PSF_xy_ without the wedge (dashed blue line). The PSF_x_ exhibits some interesting features upon introduction of the wedge device (see Fig. 3f–h, solid lines). The two most salient features are an increase in PSF_x_ compared to PSF_y_ and a dependence on the x position (direction orthogonal to the wedge interface). First, when utilizing the wedge device, the PSF_x_ for all points is significantly greater than the PSF_y_. As described above, this overall increase is likely due to chromatic aberration. In the direction of the wedge deflection, x, the wavelengths are spread out due to a wavelength-dependent index, increasing the PSF_x_ from the diffraction-limited minimum width, which is approximately PSF_y_. By dividing the green BK7 lateral shift in Table 2 by the magnification, we can obtain a rough upper bound on the increase: 1.25, 0.62, and 0.21 μm for 10, 20, and 60x, respectively. The measured increase (approximately PSF_x_ – PSF_y_ of beads near the left side of the FOV) is about 50–70% of our upper bound. The beads utilized have a peaked spectrum within the filter bandwidth, reducing the wavelength range and the increase from the diffraction limit. Second, both the PSF_x_ and PSF_z_ increase significantly as the position approaches the CCD center. Qualitatively, this increase in PSF_xz_ occurs because the effective numerical aperture of points imaged near the CCD center is reduced compared to points imaged near the CCD edges: As shown in Fig. 1d, the objective is a converging lens and refracts light originating from one side of the sample toward the contralateral side. Our device is beyond the objective back focal plane, so light originating from one side of the sample primarily transmits through the contralateral wedge and filter. In Fig. 1d, the beam of light originating from the object arrowhead primarily passes through the green filter and wedge underneath. Depending on the objective parameters, a smaller portion of the beam passes through the red filter and wedge. The opposite is true for the light beam originating from the object base (not shown). This dependence of beam size on position yields an effective decrease in the numerical aperture for points imaged near the CCD center (light originating from same side as wedge) compared to points imaged near the CCD edge (light originating from side contralateral to wedge). The beam extent and, hence, the numerical aperture in the x direction decreases, but the beam extent and the numerical aperture in the y direction does not decrease. Thus PSF_x_ and PSF_z_ increase, but PSF_y_ is roughly unchanged for points near the CCD center. We are currently quantifying the effect of various objective parameters, such as the numerical aperture and back aperture, on the PSF.

We simulated the effect of our wedges on resolution and aberration in our microscope using the Huygens calculation in Zemax. We obtained the transverse PSF of the microscope system with wedges at five locations in the FOV. As shown by the data points in Fig. 3f–h, the simulated and experimental PSF_y_ match closely. The computed PSF_x_ follow the trends seen experimentally. The intrinsic functions in Zemax are not conducive to straightforward PSF_z_ computations. Computed PSF_z_ (data not shown) were significantly greater than most experimental PSF_z_, and the trends across the FOV were less pronounced. We believe the experimental PSF_z_ is a better measure of the performance of our device.

We obtained Seidel coefficients from Zemax (see Supp. Table S1) which indicate that the aberrations from the front surface of the wedge (surface 9) are cancelled by the back surface of the wedge (surface 10). Both measured and calculated PSFs (see Fig. S2) also show minimal secondary peaks that are the hallmarks of aberration^4^. Thus, wedges introduce negligible spherical aberration, coma, astigmatism, field curvature, and distortion.

### Four-channel device

A distinct advantage of the wedge approach is easy scalability to a greater number of channels. The four-channel device deflects light toward the four CCD quadrant centers by defining two wedge parameters: a single altitudinal wedge angle set by the distance from the CCD center to the quadrant centers and an azimuthal angle set by the deflection angle to each quadrant center. The FOV of each channel is one quarter of the original FOV with no change in magnification. Each channel utilizes 25% of the available light. We fabricated the four-channel device (see Fig. 3c) by machining a BK7 window at a wedge angle of 1.55°. We quartered the circular wedge at an azimuthal angle of 0 ± 36.8° or 180 ± 36.8° to the wedge gradient to create wedge quadrants, similar to the two-channel procedure described above. We paired each quartered wedge with a quartered filter (red ET610/40 m, green ET530/20 m, blue ET470/24 m, and all three colors 69011 m) specific for imaging Brainbow, a multi-fluorescent protein technique (see below). The filter cube also contained excitation and dichroic filters from multichannel filter set 69011. All filters originated from Chroma Technologies. We adjusted the azimuthal orientation of the entire device by eye. The four-channel wedge device tiles the channels on the CCD well (see Fig. 3d), with less than 14% of the CCD area not represented in all channels. Again, precision alignment under mass production should significantly improve the performance.

### Demonstration on animals *in vivo* and fixed cell sections

**Figure 4:**
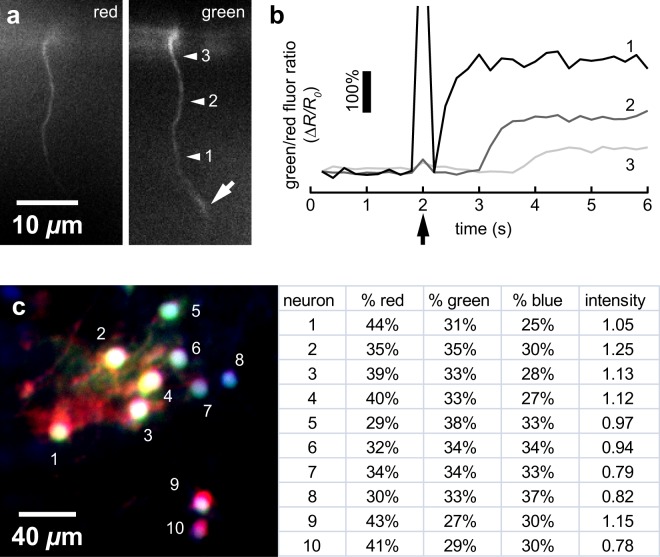
Demonstration of approach *in vivo* and in fixed samples. (**a**) Single frame (0.2 s exposure) capture of RFP (red) and GCaMP (calcium-sensitive green) channel images of D-type motor axon in *C. elegans in vivo* prior to surgery at arrow (out of focus in frame). (**b**) Relative changes in green/red ratio reveal intracellular calcium dynamics at positions 1–3. Laser axotomy occurs at *t* = 2 s. Note delay and reduction in calcium transient with distance from surgery position. (**c**) Recombined image of electroporated chick embryo expressing Brainbow. Each neuron can be distinguished by a cell-specific ratio of fluorophores and intensity (normalized values).

We used the two-channel device to measure *in vivo* intracellular calcium dynamics in multiple neurons of the nematode *C. elegans*. These neurons co-express baseline red fluorescent protein (RFP) and calcium-sensitive green GCaMP3. The green/red fluorescence ratio (*R*) specifies the relative changes in calcium levels resulting from neuronal activity or membrane poration. Figure 4a shows a single frame capture of a single axon in an intact adult animal. Figure 4b shows intracellular calcium dynamics at positions 1–3. Using a femtosecond laser, we ablated a submicrometer region of the axon at the position noted by the arrow^5^. Previous studies indicate that laser disruption of the cell membrane allows a large calcium influx into the cell and initiates an intracellular calcium wave from the damage point^6^. Accordingly, after surgery and transient laser artifact at *t* = 2 s, we observe calcium levels rapidly increase as extracellular calcium enters via the surgery site. We note a propagating calcium wave whose onset and height vary with distance from the surgery site. Our prototype device observes excellent fluorescence signal and dynamic ranges (400% change from initial value, *R*_0_) similar to those seen by conventional two-channel imaging instruments^6^.

We demonstrated our four-channel device on Brainbow samples, where neurons express a cell-specific ratio of three or more fluorescent proteins, allowing cell-specific identification of neurons and their fibers by their distinct color^7^. Brainbow imaging is typically performed by serial fluorophore excitation, often by laser scanning. This leads to differential photobleaching and extensive spectral distortion that necessitates extensive compensatory post-processing. We demonstrated the four-channel device on fixed sections of electroporated chick spinal cords expressing tdTomato (red), yellow fluorescent protein (yellow-green), and mCerulean (blue) fluorophores. Following a minimal registration and post-processing protocol described in the Methods, we successfully identified neurons individually by their spectra and intensity as shown in Fig. 4c. Thus, the four-channel device permits rapid, simple, and robust Brainbow imaging that does not require extensive post-processing.

## Discussion

In our study, we opted for a simple approach to reduce cost and complexity; however, there are many refinements that could improve performance. First, we utilized separate wedges and commercially-available filters in our prototypes to reduce cost. Depositing the thin-film sputtered filters directly onto the wedges would improve transmission, enhance durability, and reduce possible distortion. Second, the deflection, and consequently displacement on the CCD, has a small dependence on the initial angle of the light ray, *θ*_*i*_, as can be seen in equation (1) of the Supp. Note. In our study, we mounted our device in a standard emission filter housing of a filter cube, which is angled slightly from the beampath to prevent backreflections. This results in a shift of the effective incident angle, *θ*_*i*_. To minimize distortion, the filter housing and cube can be designed to orient the wedge at the angle of minimum deviation, where the dependence on *θ*_*i*_ is minimized^8^. Third, we can reduce chromatic aberration by employing substrates with decreased dispersion (*i.e*., higher Abbe number)^9^. Fused silica and CaF_2_ are two examples (see Table 2); however, not all substrates will be suitable for depositing thin-film filters. Also, similar to dispersion correction in lenses (*e.g*., achromatic doublet), we can employ two wedges of different substrates oriented oppositely to reduce the chromatic aberration at a significantly increased cost. We are currently exploring these refinements with our manufacturing partners.

In conclusion, we describe a novel method for simultaneous multichannel microscopy involving wedge prisms rather than mirrors to redirect light to the camera. We demonstrate a two-channel approach *in vivo* and a four-channel approach on fixed tissues. The simplicity and the effectiveness of our method makes it very attractive for biologists. By replacing a single emission filter, our novel device allows standard epifluorescence microscopes to perform simultaneous multichannel widefield imaging with many advantages over existing options. Our tests indicate that the devices perform as theoretically predicted, with minimal aberration but some degradation of resolution, primarily at the periphery of the FOV. Thus, the wedge prism approach represents a simple, cost-effective, robust, and user-friendly alternative to conventional simultaneous multichannel imaging.

## Methods

### Bead and dye measurements

To characterize the general operation of our devices, we imaged fluorescent beads and fluorescein isothiocyanate (FITC) dye. Fluorescent beads: we employed Fluoresbrite 1.0 μm blue-green fluorescent microspheres (Polysciences catalog #17154) and FluoSpheres 0.5 μm red fluorescent microspheres (Invitrogen catalog # F8812). We diluted bead stock solutions 1:1 v/v in 1% sodium dodecyl sulfate to reduce clumping and then diluted this mixture 1:100 v/v in ethanol to allow rapid evaporation. We spread 10 µL of the ethanol mixture onto a 15 mm × 15 mm area of a 22 mm coverslip. Beads remain fixed after evaporation. FITC dye: we dissolved 20 mg of FITC (Sigma-Aldrich F7250) in 1 mL of ethanol and mixed 50 μL of this solution in 950 μL of water. We spread 2 μL of this mixture between a slide and a 22 mm coverslip.

### Transmission measurements

It is preferable to measure transmission through an entire microscope setup with the two-channel wedge or dual-view rather than just note transmission through the devices, as they may affect downstream transmission. We computed the ratio of wedge to dual-view transmission (see Table 1) by sequentially imaging the same beads on the microscope platform with the devices and dividing their intensities as imaged by the CCD. The dual-view device employed the same emission filters and T560lpxr dichroics as the two-channel wedge device.

### Bead point spread function measurements

To measure the impact of our device on the point spread function (PSF) of various objectives, we utilized a user-friendly, automated analysis software called PSFj (http://www.knoplab.de/psfj/)^3^. Following their established protocol, we obtained z-stacks of 175 nm green beads from PS-Speck Microscope Point Source Kit (Molecular Probes P-7220) with z intervals of 500 nm (10x and 20x objectives) and 100 nm (60x objective) with and without our two-channel wedge. We masked the red channel side. PSFj fits data from the z-stacks to Gaussian functions in three dimensions, yielding three full width at half maximum (FWHM) PSF measurements, which we abbreviate as PSF_xyz_. To obtain more accurate results, we only utilized data from beads where the coefficient of determination (R^2^) for all three PSF fits is 0.95 or greater. We averaged measurements from 40 beads (the nearest neighbors in x direction) to produce each point in Fig. 3f–h.

### Zemax simulations

We simulated the PSF of the microscope with and without wedges using the Huygens PSF calculation in Zemax (OpticStudio 2017), a commonly-used optical design and simulation software. Based on the emission spectrum of the fluorescent beads, we set the simulated rays to comprise 9% 500 nm, 41% 515 nm (primary), 35% 530 nm, and 15% 550 nm light. Surfaces in the optical path are listed in Table 3. We modeled the transmission through a single wedge of a device. The wedge device consists of an offset rectangular aperture to allow transmission through one wedge only, a circular aperture, the wedge front surface (tilted at 1.24°), and the wedge back surface.

**Table 3:** Zemax model.

Surface	Type	Comment	Radius	Thickness	Material	Coating	Semi-Diameter	Mech Semi-Diameter	Focal Length	OPD Mode	Aperture Type	Aperture Max Radius
0	STANDARD		Infinity	0	N-BK7	AR	0.31	2.55	0			
1	STANDARD	cover glass	Infinity	0.17	N-BK7	AR	0.5	2.55	0			
2–6	**objective inserted here**											
7	STANDARD	rect aperture	Infinity	0.5			24.38	24.38	0		Rectangular	
8	STANDARD	circ aperture	Infinity	0.5			24.39	12.7	0		Circular	12.7
9	TILTSURF	wedge	Infinity	3	N-BK7	AR	23.62	23.62	0		Floating	
10	STANDARD	air space	Infinity	40			12	12	0		Floating	
11	STANDARD	AC254-200-A-1	77.4	4	N-SSK5	THORASLAH64	12.7	10.75	0		Circular	10.75
12	STANDARD	AC254-200-A-2	−87.57	2.5	LAFN7		12.7	10.75	0		Circular	10.75
13	STANDARD	AC254-200-A-3	291.07	194.3		THORASLAH64	12.7	10.75	0		Circular	10.75
14	STANDARD	image plane	Infinity	0			4.96	4.96	0			
**10x objective**												
2	STANDARD	working distance	Infinity	1.2			2.55	2.55	0			
3	STANDARD	front aperture	Infinity	18.69			1.85	3	0		Circular	3
4	PARAXIAL	Nikon 10x CFI Super Fluor	Infinity	0			10	11.7	20	1	Circular	11.7
5	STANDARD	objective body	Infinity	44.7			15.5	15.5	0		Floating	
6	STANDARD	back aperture	Infinity	161			10.16	6.95	0		Circular	6.95
**20x objective**												
2	STANDARD	working distance	Infinity	2.1			2.30	2.30	0			
3	STANDARD	front aperture	Infinity	7.80			1.85	1.8	0		Circular	1.8
4	PARAXIAL	Nikon 20x CFI60 Plan Fluor	Infinity	0			5	8	10	0	Circular	8
5	STANDARD	objective body	Infinity	54.65			5.82	5.82	0		Floating	
6	STANDARD	back aperture	Infinity	161			10.16	4.85	0		Circular	4.85
**60x objective**												
2	STANDARD	working distance (oil)	Infinity	0.21	N-BK7		2.68	13	0			
3	STANDARD	front aperture	Infinity	4.69	N-BK7		1.85	13	0		Floating	
4	PARAXIAL	Nikon 60x CFI Plan Apo	Infinity	0			8	13	3.33	0	Circular	13
5	STANDARD	objective body	Infinity	59.66			15.5	15.5	0		Floating	
6	STANDARD	back aperture	Infinity	161			10.16	4.2	0		Circular	4.2

Notes:

Chip zone, conic, TCE, aperture minimum radius = 0.

X, Y tangent = 0 except for wedge front surface y tangent = −0.0217.

Surface 7, rect aperture, has half width (x, y) = 12.7, 6.35; y aperture decentered 6.5.

Comments and thicknesses refer to the space after the surface described in row.

Parameters in mm.

Surfaces 7-10 comprise wedge device.

Many of the tube lens and objective specifications are proprietary. Thus, we modeled the tube lens as a 200-mm focal length Thorlabs AC254-200-A achromat. Because this achromatic lens is not flat-field corrected, the focal plane is projected onto a curved Petzval surface rather than the flat CCD. Thus for each PSF calculation we slightly adjusted the distance between the tube lens and the image plane to maximize the PSF intensity. We modeled the objectives as perfect lenses with a focal length that yields their proper magnification when paired with the tube lens. Without the wedge device in the optical path, we adjusted the back aperture of the objectives so that the simulated PSF_xy_ matched the experimental PSF_xy_. Then, using the same parameters, we calculated the PSF of the microscope with the wedge device. We extracted PSF_x_ and PSF_y_ from the Zemax data using custom code in Matlab (R2019a).

Using the same parameters as above, we generated Seidel coefficients at 515 nm wavelength by the Aberrations function of the Analyze menu.

### Imaging characteristics

The FOV is 899.3 × 671.9 μm for 10x, 446.9 × 333.9 μm for 20x, and 149.4 × 111.7 μm for 60x objectives. The camera array size is 1392 × 1040 pixels and each pixel’s size is 6.45 μm × 6.45 μm. For *in vivo* images (in *C. elegans*) we binned 2 × 2 for an effective array size of 696 × 520 pixels.

### Registration and image postprocessing protocol

Image registration is a topic that has been extensively researched over several decades comprising many involved techniques^10,11^. We registered our two-channel and four-channel devices only for simple horizontal and vertical translation of the channels. Magnification or rotation should not occur because they would require a spherical or screw surface, which is unlikely due to flat machining we employed.

For two-channel registration, we imaged typical green beads, which fluoresce brightly in the green channel and dimly in the red channel, due to the wide bandwidth of the beads. As shown in Fig. S1, we bisected the raw image of green beads and separately normalized the images, which we then registered. Registration of two-channel images from biological media is typically accomplished manually by eye or by automated software. We registered our images by eye, a combination of ImageJ and Fiji plugins (TurboReg^12^ and Descriptor-based registration^13^), and a custom cross-correlation program; comparisons did not indicate any significant difference. We utilized the translation parameters for extracting the channels from *in vivo* and fixed sample imaging. We saved the registered images both separately for quantitative analysis and together to judge colocalization by eye.

We registered four-channel images similarly but with red and blue-green beads to establish landmarks in all the channels. Under an established Brainbow postprocessing procedure^14^, we performed two normalizations to homogenize the data and enhance contrast. First, imperfectly-aligned microscope setups may have nonuniform excitation and emission transmission across the FOV. Our wedge devices typically magnify these inhomogeneities. To compensate, we normalized acquired images to an image of a uniform thin film of FITC taken under the experimental setup. Second, we linearly normalized each channel across its dynamic range in the image to enhance contrast. These normalized channels are recombined to form the final color image or generate quantitative data shown in Fig. 4c. Brainbow postprocessing seeks to highlight chromatic differences between neurons rather than preserve the original, raw ratios of the fluorescent proteins. These differences aid in pairing cell bodies and their fibers by automated systems and by eye.

## Supplementary information

Supplementary information 1

Supplementary information 2 

Supplementary information 3 
