# Supplementary material for: Wedge prism approach for simultaneous multichannel microscopy
Source: Sci Rep. 2019 Nov 28;9:17795. doi: 10.1038/s41598-019-53581-9 (PMC6882912; doi:10.1038/s41598-019-53581-9)
Supplement: Supplementary file 1 — Supplementary information 1 [file 41598_2019_53581_MOESM1_ESM.docx]

# Supplementary Note for Wedge prism approach for simultaneous multichannel microscopy. Cai, Wang, Wainner, Iftimia, Gabel, and Chung

# *Derivation of deflection angle θ_d_*

Following ref. [8], a prism of arbitrary apex angle $\alpha$ deflects rays by angle

$$\theta_{d}=\theta_{i}+\sin^{-1} \left[ \sin\alpha*\sqrt{n^{2}-\sin^{2} \theta_{i}}-\sin\theta_{i}*\cos\alpha\right]-\alpha,$$

where $n= {n_{wedge}}/{n_{air}}$.

Since $\theta_{i},\alpha\ll1$ (paraxial approximation), we can Taylor approximate the trigonometric functions. Keeping through the third order,

$\theta_{d}=\theta_{i}-\alpha+\sin^{-1} \left[ \left( \alpha-\frac{1}{6}\alpha^{3} \right)\sqrt{n^{2}-\left( \theta_{i}-\frac{1}{6}\theta_{i}^{3} \right)^{2}}-\left( \theta_{i}-\frac{1}{6}\theta_{i}^{3} \right)\left( 1-\frac{1}{2}\alpha^{2} \right) \right]$

$\theta_{d}=\theta_{i}-\alpha+\sin^{-1} \left[ \left( \alpha-\frac{1}{6}\alpha^{3} \right)\sqrt{n^{2}-\left( \theta_{i}^{2} \right)}-\left( \theta_{i}-\frac{1}{2}\theta_{i}\alpha^{2}-\frac{1}{6}\theta_{i}^{3} \right) \right]$

$\theta_{d}=\theta_{i}-\alpha+\sin^{-1} \left[ \left( \alpha-\frac{1}{6}\alpha^{3} \right)n\sqrt{1-\left( \frac{\theta_{i}}{n} \right)^{2}}-\theta_{i1}+\frac{1}{2}\theta_{i}\alpha^{2}+\frac{1}{6}\theta_{i}^{3} \right]$

$\theta_{d}=\theta_{i}-\alpha+\sin^{-1} \left[ \left( \alpha-\frac{1}{6}\alpha^{3} \right)n\left( 1-{\frac{1}{2}\left( \frac{\theta_{i}}{n} \right)}^{2} \right)-\theta_{i}+\frac{1}{2}\theta_{i}\alpha^{2}+\frac{1}{6}\theta_{i}^{3} \right]$

$\theta_{d}=\theta_{i}-\alpha+\sin^{-1} \left[ n\left( \alpha-\frac{1}{6}\alpha^{3} \right)-\frac{1}{2n}{\alpha\theta}_{i}^{2}-\theta_{i}+\frac{1}{2}\theta_{i}\alpha^{2}+\frac{1}{6}\theta_{i}^{3} \right]$

$\theta_{d}=\theta_{i}-\alpha+\left[ n\left( \alpha-\frac{1}{6}\alpha^{3} \right)-\frac{1}{2n}\alpha\theta_{i}^{2}-\theta_{i}+\frac{1}{2}\theta_{i}\alpha^{2}+\frac{1}{6}\theta_{i}^{3} \right]+\frac{1}{6}\left[ n^{3}\alpha^{3}-\theta_{i}^{3}-3n^{2}\theta_{i}\alpha^{2}+3n\alpha\theta_{i}^{2} \right]$

$\theta_{d}=n\alpha-\alpha+\frac{1}{6}n^{3}\alpha^{3}+n\frac{1}{6}\alpha^{3}+\frac{1}{2}\theta_{i}\alpha^{2}-\frac{1}{2}n^{2}\theta_{i}\alpha^{2}+\frac{1}{2}n\alpha\theta_{i}^{2}-\frac{1}{2n}\alpha\theta_{i}^{2}$

$\theta_{d}=\left( n-1 \right)\alpha+\frac{1}{6}\left( n^{2}-1 \right)n\alpha^{3}-\frac{1}{2}\left( n^{2}-1 \right)\theta_{i}\alpha^{2}+\frac{1}{2n}\left( n^{2}-1 \right)\alpha\theta_{i}^{2}$

$\boldsymbol{\theta}_{\boldsymbol{d}}\boldsymbol{=}\left( \boldsymbol{n-1} \right)\boldsymbol{\alpha+}\frac{\left( \boldsymbol{n}^{\boldsymbol{2}}\boldsymbol{-1} \right)}{\boldsymbol{6}\boldsymbol{n}}\left[ \boldsymbol{3}\boldsymbol{\alpha}\boldsymbol{\theta}_{\boldsymbol{i}}^{\boldsymbol{2}}\boldsymbol{-3}\boldsymbol{n}\boldsymbol{\theta}_{\boldsymbol{i}}\boldsymbol{\alpha}^{\boldsymbol{2}}\boldsymbol{+}\boldsymbol{n}^{\boldsymbol{2}}\boldsymbol{\alpha}^{\boldsymbol{3}} \right]$ (1)

For our two-channel experiments, *n* = 1.512, *α* = 1.24°, and -0.64° < *θ_i_* < 0.64°. The first order term is 0.64° and third order term is at most 0.0005°. This indicates that the first order approximation in equation (1) is sufficient for device design. Note that the first order approximation is independent of *θ_i_*.

# *Derivation of image displacement*

The lateral displacement at the camera is

$\Delta=d-d_{i}$

$=f_{t}\left[ \tan\left( \theta_{i}+\theta_{d} \right)-\tan\theta_{i} \right]$ by geometrical optics, and, with difference of tangents identity

$=f_{t}\left[ \tan\left( \theta_{i}+\theta_{d}-\theta_{i} \right)\left( 1+\tan\left( \theta_{i}+\theta_{d} \right)*\tan\theta_{i} \right) \right]$

$=f_{t}\left. \tan\left( \theta_{d} \right)\left( 1+\tan\theta_{i}*\tan\left( \theta_{i}+\theta_{d} \right) \right) \right.$

$=f_{t}\left. \tan\left( \theta_{d} \right)\left( 1+\varepsilon\right) \right.$, where *ε* is the relative deviation from uniform lateral displacement,

$\Delta\cong f_{t}\left. \tan\left( \theta_{d} \right) \right.$.

The error, $\varepsilon=\tan\theta_{i}*\tan\left( \theta_{i}+\theta_{d} \right)$, leads to negligible image compression and stretching in the direction of the wedge gradient. In our setup, -0.64° < *θ_i_* < 0.64° and *θ_d_* = 0.64°. Thus, *ε* is at most 0.00025 which corresponds to a shift of less than 1/10 of our camera pixel.
