# Supplementary material for: Wedge prism approach for simultaneous multichannel microscopy
Source: Sci Rep. 2019 Nov 28;9:17795. doi: 10.1038/s41598-019-53581-9 (PMC6882912; doi:10.1038/s41598-019-53581-9)
Supplement: Supplementary file 3 — Supplementary information 3 [file 41598_2019_53581_MOESM3_ESM.pdf]

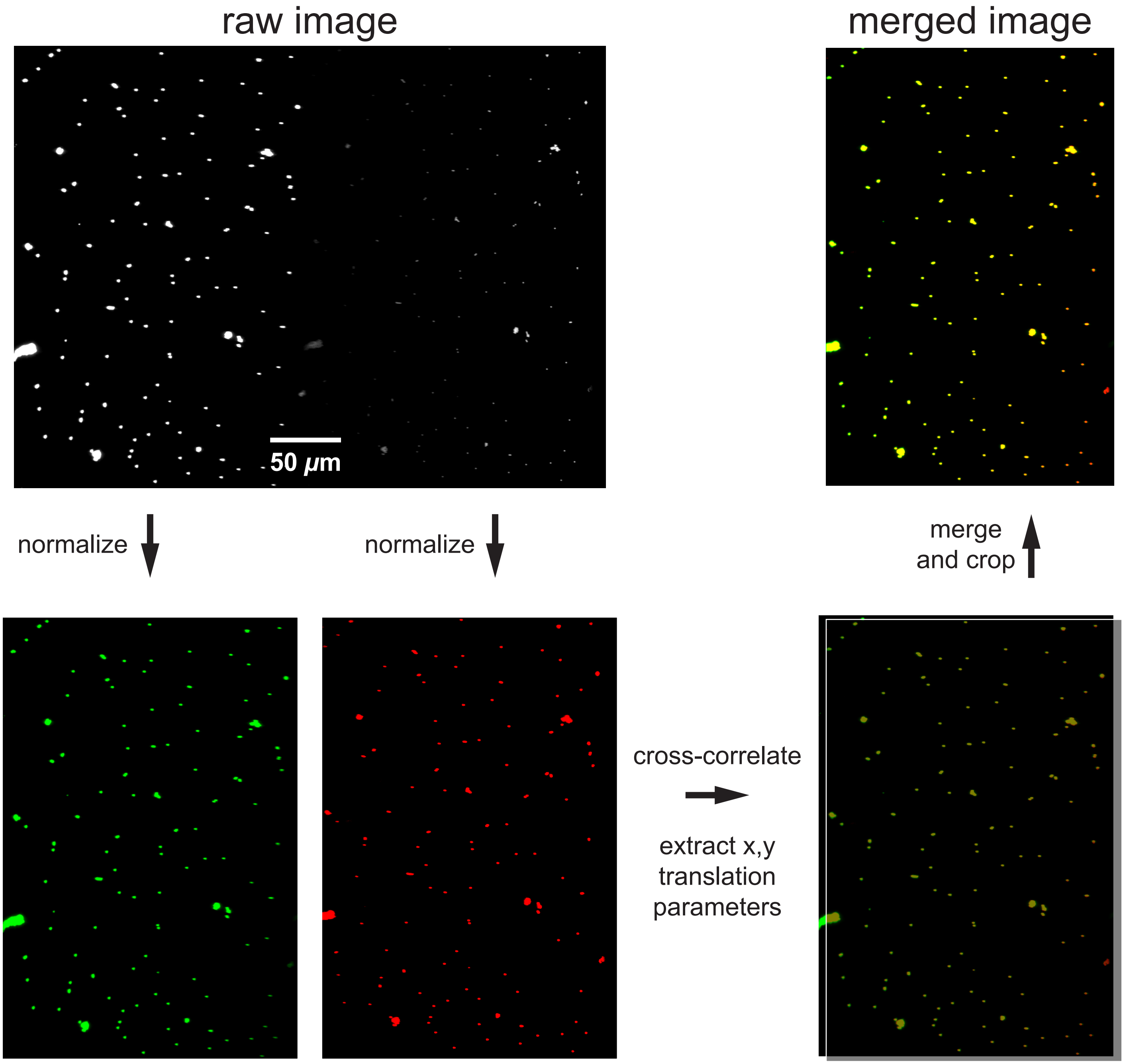

Figure S1: Registration procedure for two-channel images. We bisected and independently normalized images, extracted the translation parameters by cross-correlation, merged the images and cropped the non-overlapping regions.

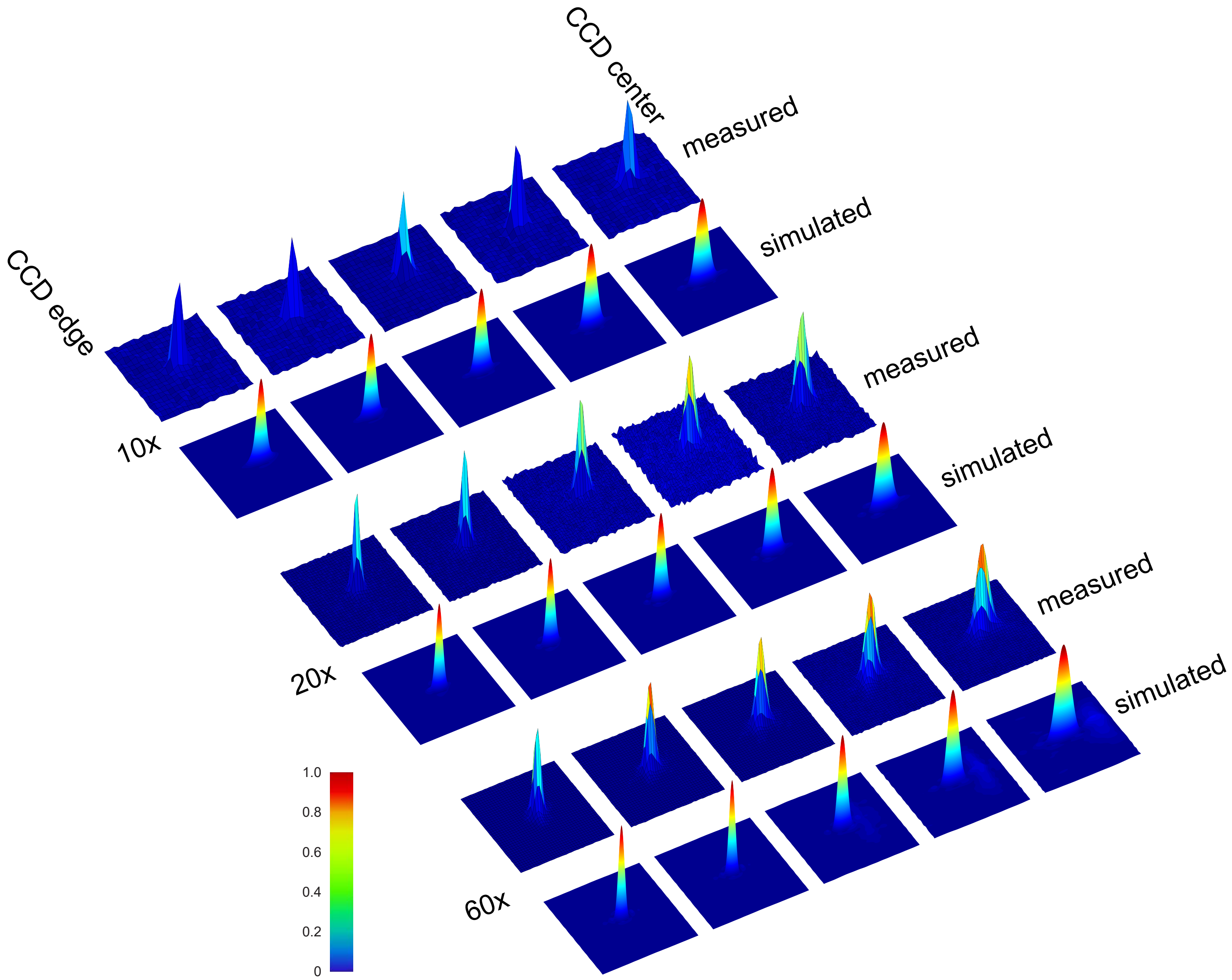

Figure S2: Measured and calculated PSFs exhibit minimal secondary peaks associated with aberrations. Transverse PSFs under various objectives were measured or simulated by Zemax. PSFs are normalized and set to same color scale. Representative PSFs from single beads are plotted.
